# The State of Exosomes Research: A Global Visualized Analysis

**DOI:** 10.1155/2019/1495130

**Published:** 2019-04-03

**Authors:** Bin Wang, Dan Xing, Yuanyuan Zhu, Shengjie Dong, Bin Zhao

**Affiliations:** ^1^Orthopedic Department, Second Hospital of Shanxi Medical University, Taiyuan, China; ^2^Arthritis Clinic & Research Center, Peking University People's Hospital, Peking University, Beijing, China; ^3^Pharmaceutical Department, Second Hospital of Shanxi Medical University, Taiyuan, China; ^4^Orthopedic Department, Yantaishan Hospital, Yantai, Shandong, China

## Abstract

**Objective:**

With the development of exosomes studies increased around the whole world. Our present study was aimed to investigate the global status and trends in exosomes field.

**Methods:**

Publications related to exosomes studies from 1994 to 2017 were retrieved from the Web of Science database. The data source was studied and indexed by using bibliometric methodology. For visualized study, VOS viewer software was used to conduct bibliographic coupling analysis, coauthorship analysis, cocitation analysis, and cooccurrence analysis and to analyze the publication trend in exosomes research.

**Results:**

A total of 4960 publications were included. The relative research interests and number of publications were increasing per year globally. The USA made the highest contributions to the global research with the most citations, the highest H-index, and the most total link strength, while Sweden had the highest average citation per item. The journal PLOS ONE had the highest publication number. The Natl Canc Ctr was the most contributive institutions. Studies could be divided into three clusters: mechanism study,* in vivo* study, and* in vitro* study.

**Conclusions:**

The efforts should be put into mechanism studies, predicted to be the next hot spots in exosomes studies.

## 1. Introduction

Extracellular vesicles (EVs), with membranous structures, are called exosomes, microvesicles, microparticles, ectosomes, oncosomes, apoptotic bodies, and many other names [1]. Among them, exosomes are biomembrane-like vesicles containing protein, miRNA, and lipids that can be delivered to the extracellular milieu (ECM) [2], with the size from 30 to 120 nm [3]. Exosomes are naturally produced within the body and could be utilized in cell-to-cell communication, molecular therapy for cancer treatment [4], and diagnosis of several skeletal disorders such as osteoarthritis [5], osteochondral regeneration [6, 7], myocardial ischemia/reperfusion (I/R) injury [8], limb ischemia, and pulmonary hypertension [9, 10].

**Figure 1:**
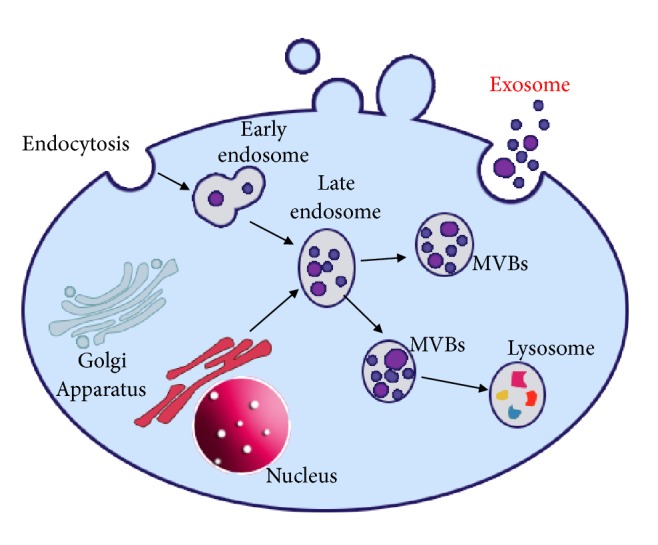
Schematic picture showing the mechanism of maturation and secretion of exosomes; there are two distinct ways: the endocytic pathway and the biosynthetic pathway. MVB: multivesicular bodies.

There are two ways to generate exosomes (Figure 1) [11]. The endocytic pathway begins with extrinsic or intrinsic signals from the local milieu. Then, the plasma membrane begins to invaginate, and the early endosome is subsequently formed. The early endosome becomes a late endosome under the regulation of multiple cell signaling pathways. The Golgi apparatus and the endoplasmic reticulum also participate in the secretion of exosomes.

It is advantageous to use exosomes concerning cell-based treatments. First, use of exosomes can avoid problems associated with the transfer of cells, which may already have damaged or mutated DNA [12]. Second, most exosomes are small and can easily circulate through capillaries, whereas the cells used in other cell-based therapies, such as MSCs, are too large to go through capillaries, and thus cannot get beyond first pass capillary beds, such as the lungs [2]. Third, the level of MSCs in cell-based therapies may quickly diminish after transplant; however, exosomes can achieve a higher “dose” than the transplanted MSCs [13]. Fourth, exosomes can also be utilized to tackle toxicity and immunogenicity problems resulting from such biomaterial treatments as nanoparticles [14]. These methods exert either positive or negative influence on regulating immune responses. Furthermore, -20°C is a suitable temperature to store exosomes [15]. However, storage destabilized the surface characteristics, morphological features, and protein content of exosomes. For preservation of the exosomes protein content and representative functional analysis, exosomes should be analysed immediately after isolation [16]. The DM SO can be used to cryopreserve the morphology of exosomal RNA but the sample quality is best characterized using fresh samples [17]. The characteristic of exosomes comparing with microvesicle and apoptotic was shown in Table 1.

In this study, publication from scientific field was treated as an indicator of the importance of that field. Information from online literature databases and metrology characteristics were analyzed through bibliometric analysis, which can be used to quantitatively and qualitatively evaluate the trends in the research community over time. Bibliometric analysis was helpful to predict development in a certain field of research by comparing the studies of main researchers, journals, institutes, and nations [18]. Besides, it also contributes a lot to clinical policy making and guidelines regulation [19]. Additionally, this feasible analysis has been applied successfully in different areas to make research studies more transparent [20, 21]. However, based on our literature review, the quantity and quality of the research on exosomes have not yet been reported. Therefore, the purpose of our study was to examine the conditions and trends of exosomes studies in different biological areas.

## 2. Materials and Methods

### 2.1. Data Source

Publication information from the Web of Science (SCI-Expanded), which was deemed as the optimal database, was analyzed via bibliometric analysis, which was introduced in previous paper [22].

### 2.2. Search Strategy

All the publication information on Web of Science was collected, and the database expiration date was set to 30 November, 2018. In our study, the research terms were as follows: theme = exosome*∗* AND publishing year = (from 1994 to 2017) AND Language = (only English) AND Document types =(REVIEW OR ARTICLE). Information about certain countries or regions was refined through region/country data on the Web of Science, where these publications come from.

### 2.3. Data Collection

The total records of those publications, including years of publications, titles, names of authors, affiliations, nationalities, keywords and abstracts, and names of publishing journals, were saved as a  .txt file from the Web of Science database and then opened by Excel 2016. Coauthors (WB and XD) separately browsed and withdrew data from these publications. Any disagreement was resolved by discussion or by asking for help from experts to reach a final consensus. Lastly, the two authors independently analyzed the data with GraphPadPrism 5.

### 2.4. Bibliometric Analysis

As was mentioned previously, the intrinsic function of Web of Science was to describe the essential features of eligible publications. The H-index is used to evaluate the impact of scientific research. The H-index assigns a value based on a scholar with an index of H having published H papers, each of which has been cited in other papers at least H times. Thus, the H-index reflects both the number of publications and the number of citations per publication [23]. The index is designed to improve upon simple measures, such as calculating the total number of citations or publications. The relative research interest (RRI) is the number of publications in a certain field divided by all-field publications per year [20].

### 2.5. Visualized Analysis

VOS viewer (Leiden University, Leiden, The Netherlands) is a software tool for constructing and visualizing bibliometric networks. These networks include journals, researchers, or individual publications, and they can be constructed based on citations, bibliographic couplings, cocitations, or coauthorship relationships. VOS viewer also offers text mining functionality, which can be used to construct and visualize cooccurrence networks of important terms extracted from a body of scientific literature [24].

## 3. Results 

### 3.1. Trend of Global Publication

#### 3.1.1. Amount of Global Publications

**Figure 2:**
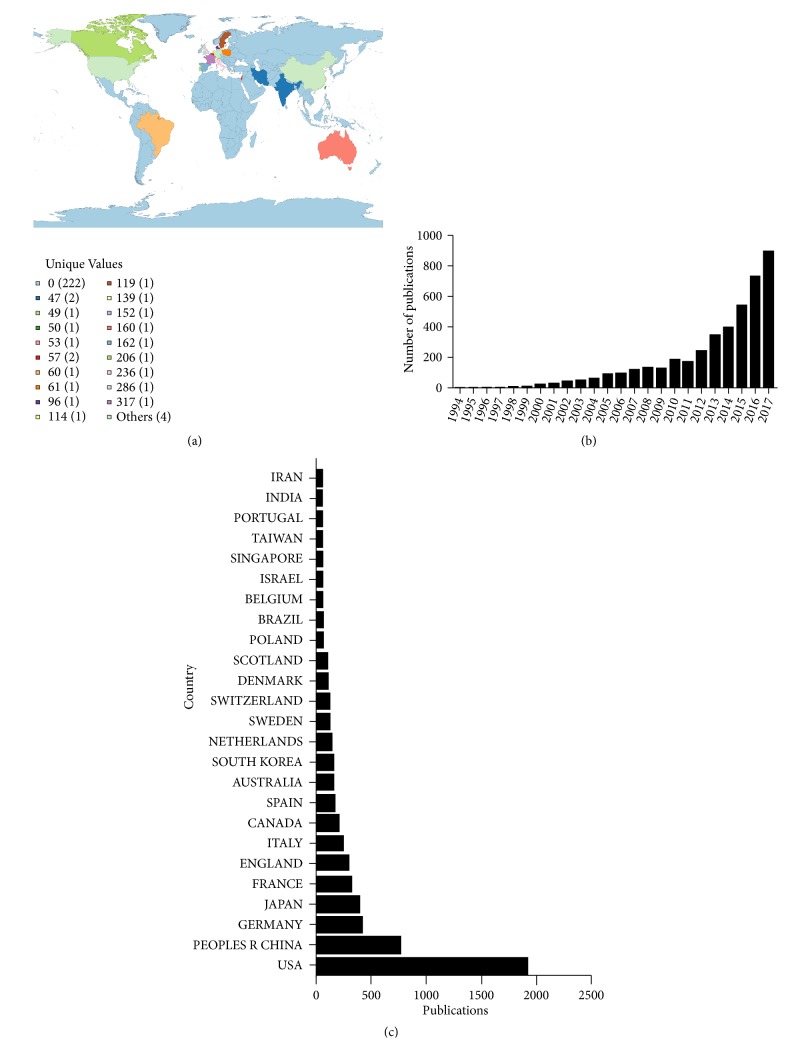
Global trends and countries contributing to exosomes. (a) The world map showing the distribution of exosomes research. (b) The single-year publication numbers in the past 23 years related to exosomes research. (c) The sum of exosomes research-related articles from the top 25 countries.

According to the search criteria, a total of 4960 articles from 1994 to 2017 were collected. When examining the amount of publications per year, most research was published in 2017 (907, 17.88%). From 1994 to 2017, we had found that there was a significant trend of global publications per year, which show the field of exosomes research was an exciting and quickly evolving area of research. (Figure 2(b)).

#### 3.1.2. Contributions of Countries

Moreover, the countries that had made the greatest contributions in exosomes research were shown in Figure 2(a). Of these included countries, USA published the most number of related articles/reviews (1949, 38.42%), followed by China (796, 15.69%), Germany (422, 8.32%), Japan (396, 7.81%), and France (323, 6.37%) (Figure 2(c)).

### 3.2. Quality of Publications of Each Country 

#### 3.2.1. Total Citation Frequency

**Figure 3:**
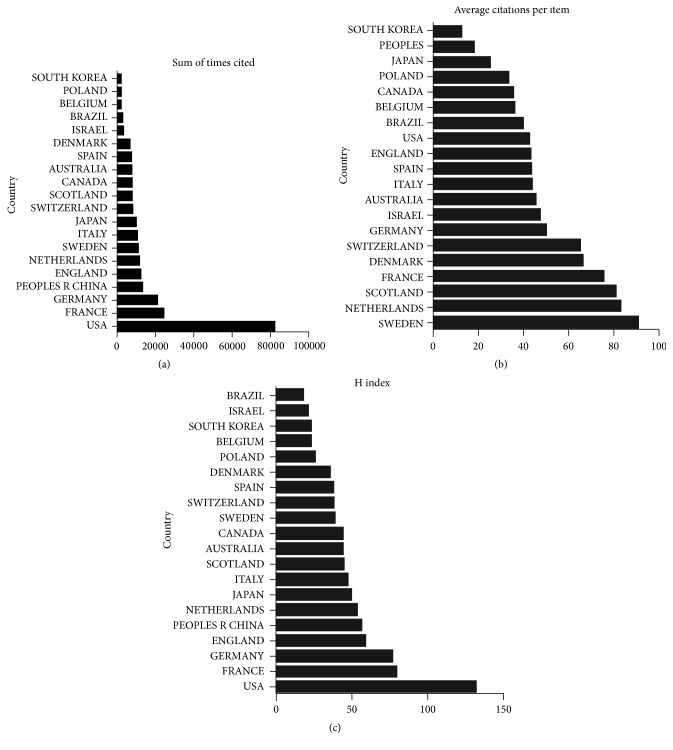
Citation frequency and H-index levels of different countries. (a) The total citations of the exosomes research articles from different countries. (b) The average citations per paper for articles from different countries. (c) The H-index of publications in the different countries.

Publications from USA had the highest number of citations (81,501), while France ranked second (23,931), followed by Germany (20,985), China (13,498), and England (12,308) (Figure 3(a)).

#### 3.2.2. Average Citation Frequency

Publications from Sweden had the most top average number of citations (90.92). The Netherlands ranked second (83.17), followed by Scotland (80.74), France (75.49), and Denmark (66.27) (Figure 3(b)).

#### 3.2.3. H-Index

The relevant publications from USA had the most top number of H-index (132), followed by France (79), Germany (77), England (59), and China (57) (Figure 3(c)).

### 3.3. Bibliographic Coupling Analysis

#### 3.3.1. Journal

**Figure 4:**
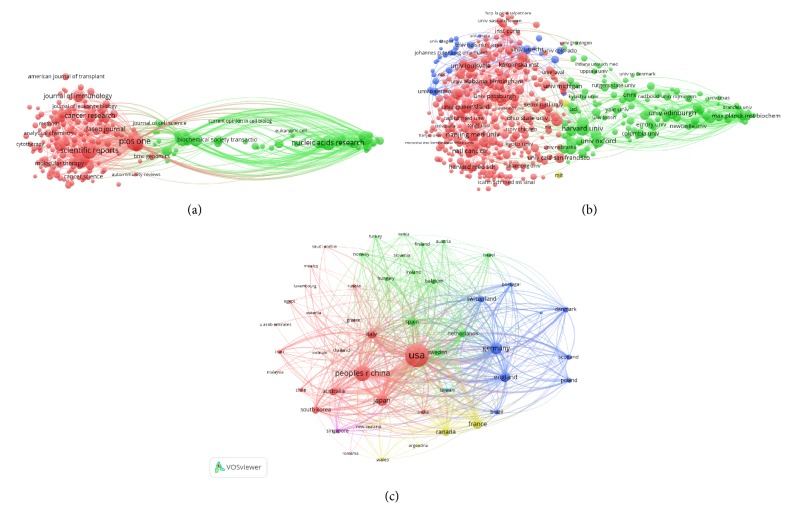
Bibliographic analysis of global research about exosomes. (a) Mapping of the 107 identified journals on exosomes. (b) Mapping of the 263 institutions on exosomes. (c) Mapping of the 43 countries on exosomes. The line between two points in the figure represents that two journals/institutions/countries had establish a similarity relationship. The thicker the line, the closer the link between the two journals/institutions/countries.

Bibliographic coupling is a similarity measure using citation analysis to establish a similarity relationship between documents. Bibliographic coupling occurs when two works refer to a common third work in their bibliographies. It is an indication that the two works probably share a related subject matter. Journal names of all publications are analyzed by VOS viewer. It is shown in Figure 4(a) that 107 journals appeared in terms of total link strength (TLS) to show the journal power. The top 5 journals having the largest total link strength were as follows: PLOS ONE (Impact Factor, IF = 2.766, 2018, TLS = 182,467 times), JOURNAL OF BIOLOGICAL CHEMISTRY (IF = 4.01, 2018, TLS = 145,905 times), SCIENTIFIC REPORT (IF = 4.122, 2018, TLS = 114,330 times), NUCLEIC ACIDS RESEARCH (IF = 11.561, 2018, TLS = 109,856 times), and JOURNAL OF EXTRACELLULAR VESICLES (TLS = 99,567 times).

#### 3.3.2. Institutions

Papers were produced from 263 institutions and were analyzed via VOS viewer, and the minimum number of documents from each organization was more than 10 (Figure 4(b)). The 5 institutions with the greatest total link strength were as follows: Natl Canc Ctr (TLS = 274,559 times), Edinburgh University (TLS = 219,990 times), La Trobe University (TLS = 219,535 times), CNRS (TLS = 219,344 times), and Harvard University (TLS = 211,842 times).

#### 3.3.3. Countries

Papers originating from 43 countries were analyzed via VOS viewer. The minimum number of documents from each organization exceeded 10 (Figure 4(c)). The 5 countries with the greatest total link strength were as follows: USA (TLS = 3,565,337 times), China (TLS = 1,705,225 times), Germany (TLS = 1,041,867 times), Japan (TLS = 950,982 times), and France (TLS = 840,987 times).

### 3.4. Coauthorship Analysis

#### 3.4.1. Author

**Figure 5:**
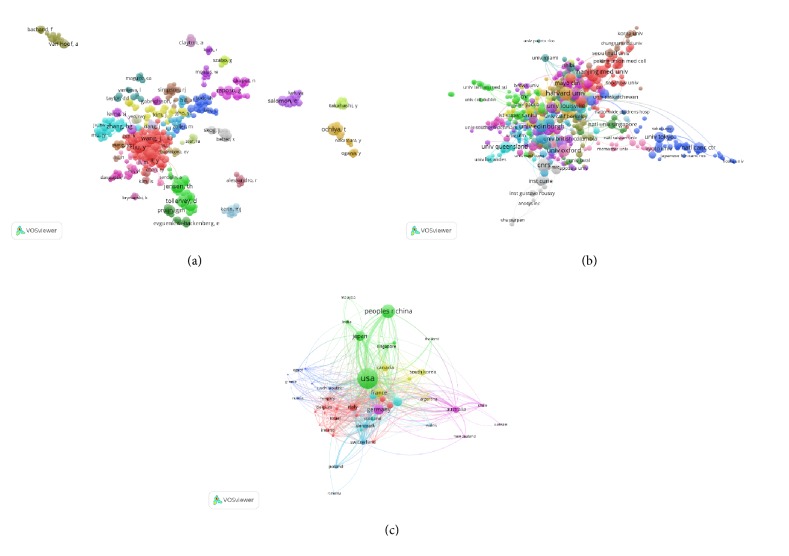
Coauthorship analysis of global research about exosomes. (a) Mapping of the 179-author coauthorship analysis on exosomes. (b) Mapping of the 263-institution coauthorship analysis on exosomes. (c) Mapping of the 43-country coauthorship analysis on exosomes. The size of the points represents the coauthorship frequency. The line between two points in the figure represents that two authors/institutions/countries had establish collaboration. The thicker the line, the closer the collaboration between the two authors/institutions/countries.

Coauthorship analysis examines the items relatedness based on the number of coauthored publications. A total of 179 authors with over 10 documents were analyzed through VOS viewer (Figure 5(a)). The 5 authors with the largest total link strength were ZHANG HG. (TLS = 196 times), ZHUANG XY. (TLS = 152 times), DENG ZB. (TLS = 133 times), MILLER D. (TLS= 112 times), and XIANG XY. (TLS = 107 times).

#### 3.4.2. Institution

Organizations with more than 10 documents were identified and analyzed through VOS viewer (Figure 5(b)). The 5 institutions with the largest total link strength were Harvard University (TLS = 175 times), CNRS (TLS = 135 times), Calif San Diego University (TLS = 127 times), Johns Hopkins University (TLS = 106 times), and Calif San Francisco University (total link strength = 105 times).

#### 3.4.3. Country

A total of 43 countries with more than 10 publications were identified and analyzed using VOS viewer (Figure 5(c)). The 5 countries with the largest total link strength were USA (TLS = 1021 times), Germany (TLS = 409 times), England (TLS = 342 times), France (TLS = 257 times), and Italy (TLS = 252 times).

### 3.5. Cocitation Analysis

#### 3.5.1. Authors

**Figure 6:**
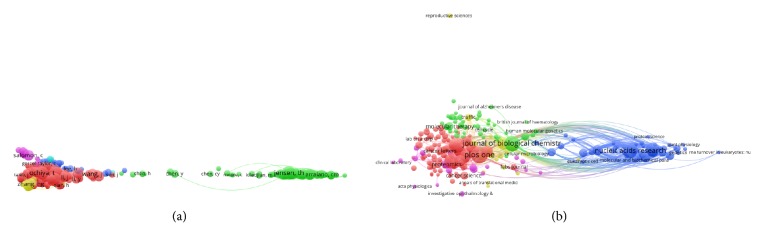
Mapping of cocitation related to exosomes. (a) Mapping of cocited author related to the field (The 1701 points with different colors represent the 1701 cited references. The size of the points represents the citation frequency. A line between two points means that both were cited in one paper. A shorter line indicates a closer link between two papers. Points in the same color belong to the same research direction). (b) Mapping of cocited journals related to the field (The 1050 points with different colors represent the 1050 identified journals. The size of the points represents the citation frequency. A line between two points means that both were cited in one journal. A shorter line indicates a closer link between two journals. Points in the same color belong to the same research direction).

Cocitation analysis considers the relatedness of items on the basis of the number of times they were cocited. There were 1701 references with more than 20 documents that were analyzed via VOS viewer (Figure 6(a)). The 5 studies with the largest total link strength were Thery C. (TLS = 51,223 times), Raposo G. (TLS = 30,654 times), Valadi H. (TLS = 27,383 times), Taylor DD. (TLS = 25,160 times), and Mathivanan S. (TLS = 20,793 times).

#### 3.5.2. Journal

The names of journals in the cocitation analysis were analyzed by means of VOS viewer. To be included in the analysis, the journals required at least 20 citations. As shown in Figure 6(b), 1050 included journals appear in the total link strength. The top 5 journals with the largest total link strength were as follows: JOURNAL OF BIOLOGICAL CHEMISTRY (TLS = 642,818 times), P NATI ACAD SCI USA (TLS = 611,986 times), CELL (TLS = 523,961 times), PLOS ONE (TLS = 481,163 times), and NATURE (TLS = 457,195 times).

### 3.6. Cooccurrence Analysis

**Figure 7:**
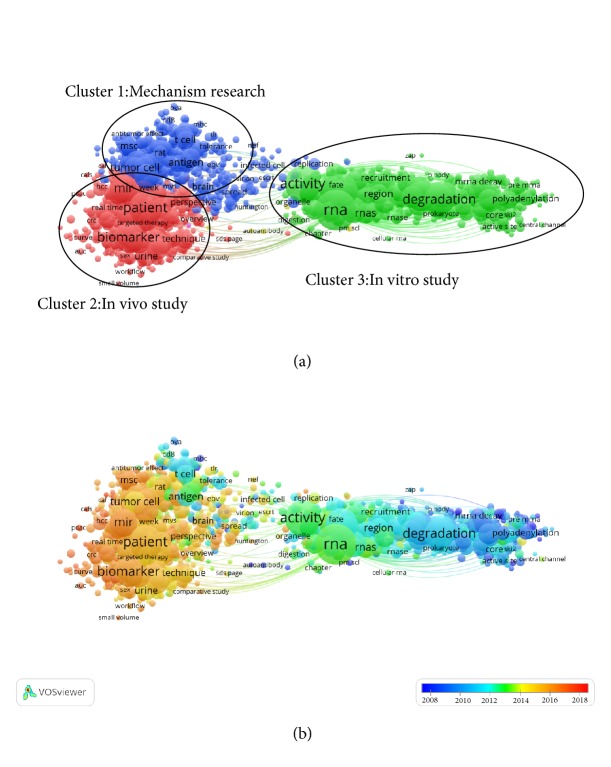
Cooccurrence analysis of global research about exosomes. (a) Mapping of keywords in the research on exosomes; the size of the points represents the frequency, and the keywords are divided into three clusters: mechanism research (left in blue), in vitro study (right in green), and in vivo study (down in red). (b) Distribution of keywords according to the mean frequency of appearance; keywords in blue appeared earlier than those in yellow and red colored keywords appeared later.

Purpose of the cooccurrence analysis is to identify popular areas and directions of researches, and it has turned out to be very important in monitoring developments in scientific areas and other disciplines. Keywords (defined as words that are used more than 5 times in titles and abstracts among identified publications) were also analyzed via VOS viewer. It was shown in Figure 7(a) that the 1603 included keywords were grouped into approximately 3 clusters: “Mechanism research”, “*In vivo* study”, and “*In vitro* study” (Figure 7(a)). These results showed the most prominent topics in exosome research so far. In the “Mechanism research” cluster, the frequently used keywords were biomarkers, circulating micrornas, microvesicles, lung-cancer, breast-cancer. For the cluster of “*In vivo* study”, the primary keywords were exosomes, stromal cells, dendritic cells, mesenchymal stromal cells, apoptosis. For the “*In vitro* study” cluster, the main keywords were exosome, messenger-rna, ribosomal-rna, saccharomyces-cerevisiae.

Keywords were noted with colors by VOS viewer based on the mean times they appear in all included publications (Figure 7(b)). The blue color indicated that the keywords appear earlier, whereas red colored keywords appear later. Figure 7(b) showed that most of the studies prior to 2014 focus on “*In vivo* study” and “*In vitro* study”. However, the recent trends showed that the third cluster, “Mechanism research”, will be extensively focused on in the future.

## 4. Discussion

### 4.1. Trends in Exosome Research

Visualized analysis and bibliometrics can be applied to describe the current status quo and to make predictions about future research. Thus, our study intends to evaluate exosome research with visualized analysis among contributing countries and institutions and predict the focus of upcoming research. In recent years, the progress achieved in the evolving field of exosomes has been exciting and quick. As shown in our study, each year witnessed significant increases in the number of publications. Furthermore, the relative research interests of publications had risen dramatically over last several years. In our study, 43 countries were shown to have published articles in this field. This indicated that more and more studies offering in-depth knowledge about exosomes would be published in the future. And our cooccurrence analysis could provide possible topics for future research.

### 4.2. Quality and Status of Global Publications

The total number of citations and H-index from one country represents the quality and academic impact of its publications. Our study shows that the USA had made the largest contribution to global exosomes research, in terms of H-index as well as total number of publications. However, Sweden had the highest average number of citations. Therefore, the USA could be regarded as the pioneer of the world in the field of exosomes research. China ranked the second in total number of publications. However, the H-index and total citation frequency of China only ranked fifth and sixth, respectively. This contradiction between the quantity of citations and quality of publications from China may be attributed to the fact that the Chinese academic evaluation system tends to focus on the quantity of publications instead of quality [25]. With the gradual increase in funds for research in China, the quality of studies from China should improve and comply with global publications in the field of exosomes research.

PLOS ONE, JOURNAL OF BIOLOGICAL CHEMISTRY, SCIENTIFIC REPORT, and NUCLEIC ACIDS RESEARCH published the most studies on exosomes. The journals in Figure 4(a) may constitute the main channel for future publications in this area.

Institutes from the top 5 countries contributed greatly to the research on exosomes, which was consistent with the publication numbers produced by the top 5 countries. Nearly all the top 20 institutes were located in the top 5 countries. This suggested the important role of first-class research institutes in improving the academic ranking of a country. The authors with the most publications in the field of exosomes research were also listed. These authors may influence the direction of future research on exosomes. Thus, their work should be given prior attention in order to obtain the latest advancements in this research.

In our study, we used bibliographic coupling analysis to set up a similarity relationship among different publications based on countries, journals, and institutions. Bibliographic coupling takes place when two articles contain citations of the same articles or journals. Those data indicated that PLOS ONE is the most closely related journal, while the USA maintained the leadership in the field of exosomes research. Coauthorship analysis is utilized to evaluate the cooperation between different countries, institutions, and authors. Results with higher total link strength indicate that the countries /institutions/authors tend to work collaboratively. Cocitation analysis aims to identify the impacts of studies by counting the number of times when cited together. Current results suggested that the fundamental studies about exosomes had the greatest total frequency of cocitation. The JOURNAL OF BIOLOGICAL CHEMISTRY was the one that having the highest citation frequency in exosomes field.

### 4.3. Research Focus on Exosomes

Based on the cooccurrence analysis, popular topics and future trends in this field of study are identified. Keywords in the titles and abstracts of the included studies undergo analysis to present a map of a cooccurrence network. Based on the cooccurrence network map (Figure 7(a)), three possible research trends were observed, including mechanism study,* in vivo* study, and* in vitro* study. While these results comply with common knowledge in this field, our research could clarify the trends of future investigation. Within the center of the cooccurrence map, as is shown obviously, such keywords as exosomes, exosome, and microvesicles, etc. have a greater weight. Thus, investment in further high-quality research evaluating exosomes within the context of these three directions is still needed.

The visualization map was similar to that cooccurrence map, while these items are noted with different colors. This method of great importance was used for monitoring the research progress. These color bars signaling different scores correspond to colors. In the overlay visualization map in Figure 7(b), colors stand for the publication years. From the results, mechanism study (red color) may become the next popular subject in exosomes research. There has been an emergence of studies involving the mechanisms of mesenchymal stem cell-related exosomes in treating diseases.

Exosomes contain a multitude of molecules, and future research on their properties and functions could develop therapeutic exosomes, which could be used in a multitargeted systems biology approach for bone repair and regeneration [11, 26]. MSCs are one of the most efficient producers of exosomes among different cell types [27]. Future studies are required to dissect the components present in exosomes and investigate their underlying mechanisms in order to treat diseases. As we discussed above, the mechanism of action of therapeutic exosomes in tissue regeneration is the hotpot, which may last for a long time. There are several theories about mechanism. First, exosomes have the proteomic potency to exert diverse effects on both humoral and cellular components of the immune system [28, 29]. Second, exosomes-mediated miRNA transfer plays an important role in disease-modulating capacity of MSCs [30]. Third, MSCs exosomes may work through a protein-based mechanism of action [31].

## 5. Strengths and Limitations

Although our study had evaluated the trends and status of exosomes research via bibliometric and visualized analysis, the analyses are hardly without certain limitations. First, only English language studies from the WoS database were included in the analysis. Non-English language literature reviews were omitted, resulting in language bias. In addition, there were differences between the present results and the real world, which triggers another sort of bias. For instance, some new high-quality publications might not attract notice due to low citation frequency. Therefore, we should routinely attach much more attention to the latest published research and particularly those non-English studies.

## 6. Conclusion

The current research shows the global directions in exosomes research. The USA becomes the major contributor of this type of research and plays a leading role in global research on exosomes. PLOS ONE has the greatest number of publications concerned about this issue. Therefore, it is not hard to predict that more studies will be published in the following years. In particular, future research will be likely to focus on the mechanisms of exosomes.

## Figures and Tables

**Table 1:** The characteristic of exosomes comparing with microvesicle and apoptotic body.

Characteristic	Size	Morphology	Protein Marker	Origin	Mechanism of discharge	Composition
Exosomes	50-120nm	Cup-shaped	Alix, Tsg101, CD63, CD9	Multivesicular Body	Exocytosis of MVBs	Protein, miRNA, mRNA
Microvesicle	100-1000nm	Heterogeneous	Selectins, integrins, CD40	Plasma Membrane	Budding from plasma membrane	Protein, miRNA, mRNA
Apoptotic Body	50-500nm	Heterogeneous	Histones	Programmed cell death	Cell shrinkage and death	Protein, DNA, miRNA, mRNA

## Data Availability

The data used to support the findings of this study are included within the article.
